# Lymphoproliferation in Inborn Errors of Immunity: The Eye Does Not See What the Mind Does Not Know

**DOI:** 10.3389/fimmu.2022.856601

**Published:** 2022-05-04

**Authors:** Saniya Sharma, Rakesh Kumar Pilania, Gummadi Anjani, Murugan Sudhakar, Kanika Arora, Rahul Tyagi, Manpreet Dhaliwal, Pandiarajan Vignesh, Amit Rawat, Surjit Singh

**Affiliations:** Department of Pediatrics (Clinical Immunology and Rheumatology), Postgraduate Institute of Medical Education and Research, Chandigarh, India

**Keywords:** immunodeficiency, inborn errors of immunity (IEI), Ig/TCR gene rearrangements, lymphoproliferation, lymphoma

## Abstract

Inborn errors of immunity (IEIs) are a group of heterogeneous disorders characterized by a broad clinical spectrum of recurrent infections and immune dysregulation including autoimmunity and lymphoproliferation (LP). LP in the context of IEI may be the presenting feature of underlying immune disorder or may develop during the disease course. However, the correct diagnosis of LP in IEI as benign or malignant often poses a diagnostic dilemma due to the non-specific clinical features and overlapping morphological and immunophenotypic features which make it difficult to treat. There are morphological clues to LP associated with certain IEIs. A combination of ancillary techniques including EBV-associated markers, flow cytometry, and molecular assays may prove useful in establishing a correct diagnosis in an appropriate clinical setting. The present review attempts to provide comprehensive insight into benign and malignant LP, especially the pathogenesis, histological clues, diagnostic strategies, and treatment options in patients with IEIs.

## Introduction

Lymphoproliferation (LP) in inborn errors of immunity (IEI) refers to persistent polyclonal, oligoclonal, or monoclonal proliferation of lymphoid cells in the clinical setting of primary immunodeficiency or immune dysregulation (1, 2). The incidence of LP in IEI varies from 0.7 to 18% (3). Typically, LP occurs during disease evolution in a patient with underlying primary immunodeficiency. However, it is difficult to assess the cases with LP as the presenting feature of IEI, posing a diagnostic challenge as there are no guidelines on the diagnosis and management of such cases. Moreover, the diagnostic and therapeutic approach toward cases with non-malignant LP in IEI is not clear. The current review will attempt to summarize the clinicopathological aspects and diagnostic approach to LP in IEI with the aim to provide an insight into early diagnosis and timely management of these cases.

## Clinical Issues

Patients with LP associated with IEI usually present at a younger age with frequent extranodal involvement as compared to their immunocompetent counterparts. Clinical symptoms are non-specific and mimic those of an infection, inflammation, or neoplasia. The clinical features are characterized by chronic or recurrent lymphadenopathy, hepatosplenomegaly, extranodal infiltration, and/or peripheral blood lymphocytosis (4). In certain immune disorders such as autoimmune lymphoproliferative syndrome (ALPS) and X-linked lymphoproliferation (XLP), LP is the predominant feature at disease onset. In a diagnosed case of IEI, it is often challenging to accurately define LP as benign or malignant as both have different therapeutic and prognostic implications. Besides, the clinical suspicion and diagnosis of immune disorder underlying LP is a mammoth task and requires expertise. The relevance of correctly diagnosing and treating benign LP in IEI lies in the fact that it may not only act as a precursor lesion of lymphoid malignancy but also may be associated with an increased risk of hemophagocytic lymphohistiocytosis (HLH) (5). Thus, in a given case an apparently innocuous LP may have a sinister connotation. Treatment and prognosis of LP in IEI depend upon the severity of the underlying immune defect and need to be assessed individually.

## Diagnostic Issues

**Figure 1:**
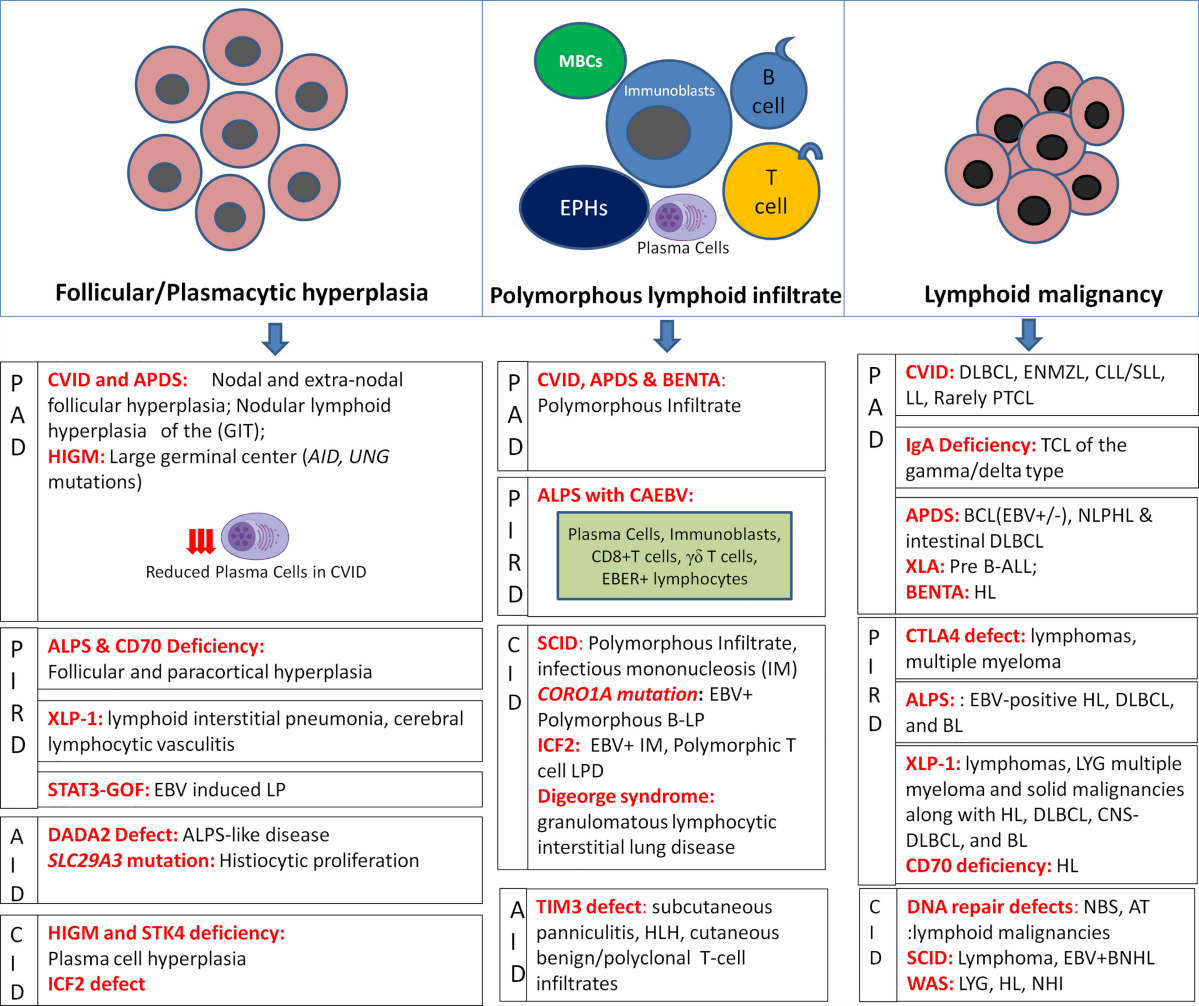
Histopathological alterations in LP associated with IEIs depicting hyperplasias, polymorphous lymphoid infiltrates, and lymphoid malignancies as per IEI disease phenotypes (MBCs, monocytoid B-cells; EPHCs, epithelioid histiocytes).

A recent nomenclature has attempted to classify LP in the setting of immunodeficiency based on the morphology of lesion, viral infection status (EBV or HHV-8), and the clinical immunodeficiency state (2). With respect to LP in IEI, the morphological spectrum chiefly encompasses Epstein-Barr virus (EBV)-associated B-cell LPDs similar to the histological lesions observed in post-transplant lymphoproliferative disorders (PTLD). These include B-cell lymphoid hyperplasias: follicular, plasmacytic, and infectious mononucleosis (IM)-like; polymorphous B-cell LP; indolent lymphomas; aggressive non-Hodgkin lymphomas (NHL); and classical Hodgkin lymphoma (HL)- like LP (2). In general, T/NK-cell LPs are rare in IEI, the majority being benign/reactive CD8+T-cell infiltrates. Certain morphological clues in tissue biopsies may help to predict the underlying immune disorder **[** **Figure 1****]**. In a recent review investigating benign and malignant LP, distinct histopathological alterations in the distribution of CD4+ follicular helper T-cells, follicular dendritic cells, and mantle zone naïve B-cells correlate with the different patterns in the development of germinal centers (6). Nevertheless, achieving a specific diagnosis is often challenging owing to the significant degree of morphological and immunophenotypic overlap between the benign and malignant lesions such as polymorphous B-cell LP is a masquerader of Hodgkin lymphoma (HL) and benign CD8+T-cell/histiocytic infiltrates in common variable immunodeficiency (CVID) could be easily confused with mimicking T- large granular lymphocytic leukemia (T-LGLL) leading to overtreatment.

## Pathophysiology and Histopathological Alterations

### Combined Immunodeficiency Disorders

In severe combined immunodeficiency (SCID), loss of T/NK cell-mediated immune surveillance leads to uncontrolled EBV-driven B-cell LP that may progress into lymphoma (7). Homozygous and hypomorphic missense mutations in *CORO1A* may present as early-onset EBV+B-LP as a result of CD4+CD45RA+ T-cell lymphopenia and impaired invariant NKT-cell development and survival defects (8). Dedicator of cytokinesis-8 (DOCK8) deficiency/hyper IgE syndrome (HIES) leads to impaired NK-cell function and increased predisposition to EBV+ lymphomas (9). In hyper IgM syndrome (HIGM) with *CD40LG/CD40* mutations, the defective antibody response to antigens results in the malignant transformation of B-cells (10). Serine/threonine-protein kinase 4 (STK4) deficiency, has been associated with nodal and extra-nodal EBV+ LP and B-cell lymphoma suggesting the role of EBV infection in inducing LP (11). Inducible tyrosine kinase (ITK) deficiency is associated with progressive CD4+T-cell and NKT-cell lymphopenia, and hypogammaglobulinemia resulting in EBV viremia and immune dysregulation leading to massive LP (12).

Histopathological changes in SCID comprise polymorphous LP characterized by systemic proliferation of highly polymorphous B-lymphoid cells showing plasmacytoid and immunoblastic differentiation that may also progress into HLH and EBV+B-NHL (1). EBV-encoded RNA (EBER)+ polymorphous CD20+ B-cell LP and DLBCL have been reported in patients with hypomorphic *CORO1A* mutations (8). DOCK8 deficiency has been reported to manifest as young-onset EBV+ and EBV- lymphomas and EBV+ pulmonary lymphomatoid granulomatosis (LYG) (9). In HIGM, the absence of germinal centers in lymph nodes and a massive extranodal accumulation of plasma cells that secrete IgM, particularly in GIT, to T-LGLL, HL, and EBV+B-NHL have been reported (1). STK4-deficiency is associated with plasma cell hyperplasia and EBV+ B-cell polymorphous LP, and B-cell lymphomas showing plasmacytic differentiation (11). ITK deficiency is chiefly associated with EBV+B-LPD, especially HL (12)

#### Combined Immunodeficiencies With Associated or Syndromic Features

Wiskott-Aldrich syndrome (WAS) patients have an increased predisposition to develop EBV-driven LYG, NHL, and HL usually following a long duration of non-malignant LP due to defective immune surveillance against the virus-infected cells (13). Patients with DNA repair defects, Nijmegen breakage syndrome (NBS), and ataxia-telangiectasia (AT) show a high predisposition to lymphoid malignancies at a young age as a result of genomic instability, chromosomal abnormalities, combined B- and T-cell immunodeficiency, and radiation hypersensitivity (14, 15). Histologically, WAS is associated with EBV+ extranodal clonal angioinvasive B-LP (LYG), HL (nodular sclerosing and lymphocyte depleted), and B-NHL, particularly DLBCL (1). Notably, T-NHL, T-acute lymphoblastic leukemia/lymphoma (T-ALL/LBL), and clonal non-malignant T-cell proliferations are more common than B-NHLs in AT, and NBS (1). An increased propensity for T-prolymphocytic leukemia (T-PLL) has been reported in children with AT (1). Among B-cell lymphomas, classical HL, diffuse large B-cell lymphoma (DLBCL), and Burkitt lymphoma (BL) are commonly described (1). Immunodeficiency with centromeric instability and facial anomalies type 2 (ICF2) is a DNA-methylation disorder with a high susceptibility to EBV infection manifesting as EBV+ IM, HLH, chronic active EBV infection (CAEBV), HL, large B-cell lymphoma, and polymorphic non-clonal T-cell LPD (16). Patients with Di-George syndrome may present with CD8+ granulomatous cutaneous T-cell lymphoma and granulomatous lymphocytic interstitial lung disease (GLILD) characterized by ill-defined non-necrotizing lymphohistiocytic granulomas, CD20+ B-cell-rich lymphoid nodules, CD4+ T-cell-rich interstitial pneumonia, and peribronchiolar follicular hyperplasia with reduced regulatory T-cells (Tregs) (17). Homozygous Post-meiotic segregation 2 (*PMS2*) mutations, a DNA mismatch-repair defect is characterized chiefly by poor antibody responses and low B-cell number and is associated with B- and T-cell leukemia/lymphoma besides colorectal cancer and brain tumors (18). The other CIDs associated with increased risk of lymphomas include Bloom syndrome, ligase 1 and MCM4 deficiencies, and cartilage hair hypoplasia (19).

#### Predominantly Antibody Deficiencies

In CVID, LP is one of the prominent clinical features accounting for approximately 20% of cases (20). Lymphadenopathy has been associated with the expansion of transitional B-cells (21). A recent report based on the United States Immunodeficiency Network (USIDNET) registry reported lymphoma in 8% of 1091 CVID patients (22). Lymphoma is the second major cause of mortality in CVID after chronic lung disease (23). B-cell lymphomas arise chiefly from germinal center-experienced mature B-cells that have undergone somatic hypermutation of Ig genes, correlating with a higher frequency of DLBCL, extranodal marginal zone lymphoma (ENMZL), and HL in CVID (24). Granulomatous CVID has been reported to involve every organ system occurring in 8-20% of CVID cases (25). The prevalence of LP in X-linked agammaglobulinemia (XLA) is extremely low (approximately 0.7%) in comparison to other IEIs (26). Nevertheless, the presence of lymphadenopathy in XLA is considered a matter of concern as it may harbor a lymphomatous process. Patients with selective IgA deficiency are usually asymptomatic, although may rarely develop infections, autoimmune disorders, and malignancies (27, 28). Considering the shared genetic basis between CVID and selective IgA deficiency, a higher incidence of malignancies has been observed, primarily gastrointestinal (29, 30). Activated phosphoinositide 3-kinase delta syndrome (APDS) is chiefly characterized by recurrent respiratory tract infections, CAEBV, immune dysregulation, and recurrent or persistent LP presenting as lymphadenopathy, splenomegaly, mucosal nodular lymphoid hyperplasia, and lymphoma (31). CAEBV results from impaired NK and CD8+ T cell-mediated killing of infected cells by EBV. Persistent EBV infection and B-cell proliferation due to constitutive activation of B-cell intrinsic PI3Kδ signaling contribute to lymphomagenesis in APDS (32). B-cell expansion with nuclear factor kappa-light-chain-enhancer of activated B cells (NF-κB) and T-cell anergy syndrome (BENTA) is a rare disorder featured by constitutive activation of the NF-κB signaling pathway leading to EBV-driven polyclonal B-cell LP manifesting as lymphocytosis, splenomegaly, and lymphadenopathy (33). NFKB1 deficiency is a clinically heterogeneous PAD with impaired function of B-cells with or without T-cell dysfunction and is characterized by recurrent infections, cytopenias, and EBV+ B-LP (34). Defects in activation-induced cytidine deaminase (*AID*) and uracil DNA glycosylase (*UNG*) in HIGM are frequently associated with lymphoid hyperplasia (35).

The morphological spectrum of LP in CVID is heterogeneous ranging from benign follicular hyperplasia and paracortical expansion with EBV-positive B-cells including large pleomorphic Reed-Sternberg (RS)-like cells to clonal but non-malignant B-cell nodular lymphoid hyperplasia of the gastrointestinal tract with a near-total absence of plasma cells to DLBCL, ENMZL, CLL/SLL, LPL, HL and rarely peripheral T-cell lymphoma (PTCL) (1). Notably, CD8+ cytotoxic T-cell proliferations are common in peripheral blood and hepatic sinusoids of CVID patients which is often difficult to differentiate from T-LGLL (36). Precursor B-ALL has been reported in XLA (37). Though rare, a few case reports have described T-cell lymphoma of the γδ type in selective IgA deficiency (28). APDS is frequently associated with non-malignant gastrointestinal and respiratory nodular mucosal lymphoid hyperplasia. These lesions show atypical follicular hyperplasia with disrupted and hyperplastic germinal centers, attenuated mantle zones, and perisinusoidal aggregates of monocytoid B-cells (31). There is hyperplasia of CD4+PD1+ follicular T-helper cells and PD1+CD57+CD8+ senescent T-cells and IgM+ plasma cells with reduced IgG+ plasma cells. EBV-associated oligoclonal polymorphous- B-cell LP has also been reported comprising polymorphous lymphoid infiltrate comprising of B- and T-cells, plasma cells, epithelioid histiocytes, and monocytoid B-cells. APDS shows an increased predisposition to EBV+/- B-cell lymphomas including intestinal DLBCL, and nodular lymphocyte-predominant Hodgkin lymphoma (NLPHL) (32). A dual clinicopathological presentation of APDS with early-onset HLH followed by HL has also been reported (38). EBV+ polymorphous B-LPD and HL are described in BENTA disease (33). NFKB1-deficient patients may present with generalized lymphadenopathy with EBV+ reactive lymphoid hyperplasia of increasing clinical severity (34). *AID* and *UNG* mutated HIGM shows enlarged lymph nodes with large germinal centers (19, 35).

#### Diseases of Immune Dysregulation

In ALPS, homozygous mutations in *FAS* and *FASL* genes are associated with impaired cytotoxicity and severely reduced activation-induced cell death (AICD) in B- and T-cells resulting in clinically severe disease and massive LP including lymphomagenesis (39). Besides ALPS, *Caspase-8* mutations may also present as end-organ LP, granulomatous inflammation, mesenteric lymphadenopathy, and recurrent EBV infection (40). CD25, CD122, cytotoxic T-lymphocyte–associated antigen 4 (CTLA-4) deficiencies, and signal transducer and activator of transcription 3 (*STAT-3*) gain-of-function mutations lead to impaired Treg function leading to impaired suppression of effector T cells causing immune dysregulation with autoimmunity and may present with LP with recurrent EBV infections (19, 41). The estimated risk of malignancies in affected patients with CTLA-4 deficiency is approximately 12.9% (41). CTLA4 insufficiency and biallelic *LRBA* mutations share similar clinical features and are also associated with granulomatous lymphocytic interstitial lung disease (GLILD) (17, 42). Elevated STAT3 signaling leads to defects in phosphorylation of STAT5 and STAT1, impaired Treg function, enhanced T-helper cell-17 differentiation, LP, and systemic autoimmunity (19, 42). XLP1 and 2 are associated with EBV-driven HLH and LP due to severely reduced iNKT cells, although XLP1 shows an increased risk of lymphomagenesis (19). Another defect in CTP synthase 1 (*CTPS1*) also leads to fatal viral (EBV) infections which results in LP and non-Hodgkin B cell lymphoma (19, 43). Similarly, RAS guanyl-releasing protein 1 (*RASGRP1)* deficiency has been involved with T-cell lymphopenia and EBV-associated B-cell lymphoma (44). Accelerated loss of TCR repertoire diversity has been observed in these cases. Additionally, CD70, CD137(4-1BB), CARMIL2, PRKCD, and CD27 deficiencies and X-linked magnesium EBV and neoplasia (XMEN) exhibit unique susceptibility to EBV infection and associated LP as a result of impaired T-cell activation and proliferation (19). Impaired CD27-CD70 interactions lead to loss of CD4+ and CD8+ T-cell response, impaired EBV killing, and CAEBV with hypogammaglobulinemia (45). Interestingly, CD70 deficiency may clinically mimic periodic fever syndrome (45). Fanconi anemia-associated protein 24 (FAAP24) deficiency, a familial HLH syndrome, results in fatal EBV-driven LP due to the failure of cytotoxic CD8+T cells to kill EBV-infected B-cells (19).

Histopathological features in ALPS include marked proliferation of non-clonal CD4-/CD8-CD45RA+ CD57+TCRαβ+ cytotoxic (TIA+ and perforin+) double-negative T-cells (DNTs), involving peripheral blood, lymph nodes, liver, spleen, and extranodal sites, a few cases may show γδ-T-cell proliferation (36, 46). Follicular and paracortical hyperplasia and PTGC have been frequently described (1). HL, classical and NLPHL, DLBCL, Rosai-Dorfman disease (RDD), and rarely PTCL have been well-described in ALPS (1). Histopathological features of LP in ALPS may overlap with CAEBV, especially in those cases where classic ALPS morphology is not evident. In such cases, florid paracortical hyperplasia comprising plasma cells, immunoblasts, EBER+ lymphocytes, cytotoxic T-cells, and γδ-T-cells is the predominant finding (46). CTLA-4 deficient patients have an increased risk of solid and lymphoid malignancies including lymphomas and multiple myeloma. The reported cases are mostly EBV-positive HL, DLBCL, and BL (41). XLP1 associated LP ranges from non-malignant LP including lymphoid interstitial pneumonia, LYG, cerebral lymphocytic vasculitis, HLH, and severe IM, to malignant lesions like HL, DLBCL, CNS DLBCL, and BL (47). Patients with 4-1BB deficiency have been diagnosed with EBER+ HL and CD20-CD38+ DLBCL on lymph node biopsies (48). CD70 deficiency can show varied presentations ranging from EBV+IM, reactive follicular and paracortical hyperplasia to HL (45). CD27 deficiency manifests as EBV+HLH and lymphomas (19).

#### Autoinflammatory Disorders

Patients with T-cell immunoglobulin and mucin domain-containing protein 3 (TIM3) deficiency usually present with subcutaneous panniculitis, HLH, cutaneous benign/polyclonal T-cell infiltrates, and panniculitis-like T-cell lymphoma of subcutaneous tissue and mesenteric fat (49). Deficiency of adenosine deaminase 2 (DADA2) is a recently described autoinflammatory disorder that is caused by bi-allelic mutations in the *CECR1* gene encoding adenosine deaminase 2 (*ADA2*). Around 30% of cases present with splenomegaly and 10% present with lymphadenopathy. DADA2 may have varied clinical presentations ranging from vasculopathy, chronic liver disease, immune cytopenias, immunodeficiency, hypogammaglobulinemia, and LP (50). LP in DADA2 includes T-LGLL, ALPS-like disease, and HL. Germline mutation in the *SLC29A3* gene causes histiocytosis-lymphadenopathy plus syndrome which is characterized by abnormal histiocytic proliferation and accumulation in lymph nodes, liver, spleen, GIT, CNS, skin, and kidneys causing organ damage (19).

#### Phenocopies of PID

Somatic activating mutations in *NRAS* and *KRAS* genes may produce an ALPS-like phenotype, Ras-associated autoimmune leukoproliferative disorder (RALD) that is featured by chronic non-malignant LP, autoimmune cytopenias, monocytosis, and hypergammaglobulinemia. LP is usually indolent, although myeloid malignancies have been reported in a few cases (19, 51).

### Laboratory Diagnosis

#### Morphological Assessment

Histopathological changes of LP often correlate with the underlying IEI. However, the pathologist should be aware of the potential diagnostic pitfalls while analyzing tissue biopsies, especially CD8+ cytotoxic T-cell infiltrates and polymorphous B-cell LP that could easily be mistaken for lymphoma leading to aggressive therapy. It is critical to understand that in IEIs, a major proportion of LP is constituted by polyclonal/oligoclonal, benign/reactive lymphoid infiltrates and every case is not a lymphoma, although the risk of malignant transformation is higher as compared to immunocompetent individuals. Detection of EBV encoded small RNA by *in-situ* hybridization (EBER-ISH), EBV encoded nucleic antigen (EBNA) and latent membrane protein (LMP) in lymphoid cells correlates well with EBV-infection and LP. EBV infection may cause downregulation of B-cell markers (CD20, CD79b, and CD19) and upregulation of CD30 (1). Immunohistochemistry (IHC), although used routinely for the detection of B-, T- cell, histiocytes, and plasma cell lineage antigens, should be interpreted with caution and in an appropriate clinical context. The tumor cells express high levels of programmed death-ligand 1 (PDL1) in CVID associated EBV+ DLBCL and HL (24). Immune checkpoint inhibitors are used to block this immune checkpoint protein, PDL1 expressed by tumor cells in HL to augment the T-cell mediated immune response. Although rare, it is difficult to diagnose T-cell lymphomas in IEIs other than DNA-repair defects owing to a high frequency of reactive/non-malignant T-cell LP. In such cases, a comprehensive approach including clinical phenotype, size of lymph node/lesion, architectural effacement, cellular/nuclear atypia, TCR gene rearrangements, chromosomal aberrations, and immunophenotyping should be followed (36).

#### Flow Cytometry

Flow cytometric *in situ* hybridization (Flow-FISH) is a powerful diagnostic tool for EBV+LP as it can quantify and phenotypically characterize the EBER-positive lymphocytes (52). In CVID, flow cytometry-based detection of an increased proportion of CD21^low^ B-cells and transitional B-cells (CD38+IgM+) and decreased proportion of class-switched memory B-cells correlates with splenomegaly, granulomatous inflammation, and lymphadenopathy (53). Several fluorescent-labeled antibodies against B/T/NK-cell lineage markers are routinely used to differentiate between reactive versus neoplastic populations. Kappa/lambda ratio (>3 or <0.5) is a more reliable indicator of the clonal process, although the complete absence of light chain expression has been described (54). T-cell receptor-Vβ (TCR-Vβ) repertoire assay is used to assess clonality in suspected T-cell LP covering almost 70% of the TCR repertoire. It is recommended for peripheral blood samples only as it gives inconclusive results with tissue samples and bone marrow aspirates (54). Nevertheless, flow cytometry is an invaluable and robust test with an accuracy of 70-90% in LPDs (54) **[** **Supplementary Table 1****]**.

#### Molecular Assays

Molecular assays used to assess clonality in suspected LP include Southern blot hybridization (SBH), restriction fragment length polymorphism (RFLP), and polymerase chain reaction (PCR)-based assays like fragment length analysis using capillary electrophoresis (Gene scan) and next-generation sequencing (NGS). These assays are largely based on detecting clonal *Ig/TCR* gene rearrangements that are random recombination events between one of several *V,* (*D*), and *J* segments generating unique exon sequences encoding antigen-bearing sites on Ig or TCR molecules for each lymphocyte. These events occur during the initial B- and T- cell development and impart diversity to Ig/TCR molecules (up to 10^12^) expressed by each lymphocyte. When identical sequences are shared by cells, it represents the clonal nature of that population, and identification of such homogeneous/clonal or heterogeneous/polyclonal populations forms the basis of clonality testing in suspected LP. A standardized and optimized multiplex PCR has been developed that targets nearly all *Ig/TCR* (*IgH, IgK, TCRγ, TCRβ, and incomplete IgH D-J and TCRβ D-J*) rearrangements by fragment length analysis (55). Due to the remarkably increased rates of clonality detection in B- and T-cell malignancies, these multiplex PCR assays have become gold-standard for clonality testing in LP. The interpretation is based on the identification of specific patterns of peaks generated by Genescan classified as clonal, polyclonal, pseudoclonal, multiple products, and non-evaluable (55). Although applicable in more than 95% of cases for routine clonality diagnostics across multiple centers, these assays have their own pitfalls, particularly when low-intensity clonal signals are generated (55). Moreover, they do not provide information about the clone-specific sequence and need to be run in duplicate for reproducible results. With the advent of NGS technology, *Ig/TCR* rearrangements may be targeted with higher sensitivity and improved clonality detection rate. The advantages of NGS over other molecular assays include a) improved detection of clonal populations in clinical samples with lymphoid cells undergoing somatic hypermutation b) the patient-specific index clone could be accurately sequenced and monitored for clonal evolution c) the entire spectrum of gene rearrangements in a sample could be visualized simultaneously, and d) clonal association between different lesions may be determined (56). Amplicon-based and targeted NGS-based strategies have been established to detect rearrangements and translocations including *IGH, IGK, IGL, TRA, TRB, TRG, and TRD* genetic loci in clinical samples (57). The targeted NGS panels are more economical, with less sample requirement, and multiplexing in a single assay, and enable uniform reporting of results along with the potential to detect copy number variations (CNV), translocations, somatic mutations, and indels associated with LPDs, thus achieving a sensitivity of 99.6% (57).

**Figure 2:**
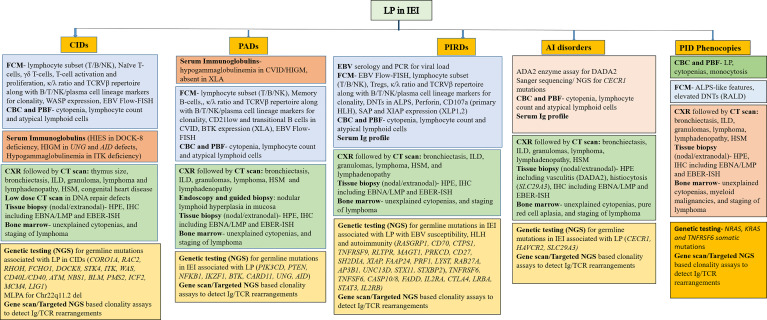
Diagnostic algorithm of investigations for LP in IEI according to clinical phenotype (FCM, flow cytometry; CBC, complete blood count; PBF, peripheral blood film; CXR, chest X-ray; CT, computed tomography; HPE, histopathological examination; NGS, next-generation sequencing; PCR, polymerase chain reaction; IHC, immunohistochemistry; EBER-ISH, Epstein-Barr virus-encoded RNA- *in-situ* hybridization; MLPA, multiplex ligation-dependent probe amplification; FISH, fluorescence *in-situ* hybridization).

Despite the availability and recent advancements in diagnostic techniques, many patients still remain undiagnosed and are either undertreated or overtreated. Being a complex disorder, it is important to understand the clinical phenotype and pathophysiology of LP in IEIs. We attempt to propose a diagnostic algorithm of investigating patients with LP with regard to specific IEIs (**Figure 2**).

#### Treatment

Improved survival with allogeneic reduced-intensity conditioning-hematopoietic stem cell transplant (RIC-HSCT) has been achieved in patients with IEI and B-cell lymphoma. These patients are treated with conventional chemotherapy and anti-CD20 monoclonal antibody (Rituximab) to achieve remission prior to the transplant. EBV infection in the pre-and post-transplant setting mandates initiation of therapy [Rituximab or EBV-cytotoxic T-lymphocytes (EBV-CTLs)] (58). HSCT is the definitive mode of therapy for many IEIs with LP including SCID and WAS. Apart from this, there are targeted therapies for some of the IEIs. Though management of CVID relies on long-term immunoglobulin replacement therapy (IVIg) and antibiotic prophylaxis, surveillance for complications is important (59). Follow-up for complications including malignancies, lymphoma, and non-neoplastic LP is important followed by appropriate therapy (22, 60).

Children with APDS show a significant reduction in infections on IVIg. HSCT with medium- or reduced-intensity conditioning has shown effective results (31, 61, 62) Autoimmune manifestations of APDS require immunosuppressive therapy (63). Rituximab has proven beneficial in the management of non-neoplastic LP. Inhibition of the downstream mTOR pathway by Sirolimus or Rapamycin decreases both non-neoplastic and neoplastic lymphoproliferation including regression of cutaneous T-cell lymphoma (31). In the European society for immunodeficiencies (ESID APDS) registry, a significant benefit (19/25) in the non-neoplastic LPD was seen on therapy with Sirolimus (64). Also, direct inhibition of the activated PI3Kδ by selective PI3Kδ inhibitors such as Leniolisib has shown a decrease in lymph node and spleen size in APDS (65).

In ALPS, treatment depends on manifestations and complications. Autoimmune manifestations, especially cytopenias require immunosuppressive therapy with corticosteroids and IVIg followed by steroid-sparing agents (66). The two most common first-line steroid-sparing agents used currently are mTOR inhibitor (Sirolimus) and Mycophenolate mofetil (MMF) (66–68). Long-term studies have shown durable improvement in lymphadenopathy and splenomegaly within 3 months of initiating sirolimus (69, 70). Patients with lymphadenopathy need to be closely monitored for lymphoma development. Severe and refractory cases require HSCT.

The definitive treatment of XLP is HSCT. However, therapy needs to be tailored according to the symptoms and infection profile. IVIg replacement therapy is essential in almost all patients to reduce infection risk and treat hypogammaglobulinemia. EBV+ patients need therapy with Rituximab. HLH needs aggressive therapy with high dose IVIg, Rituximab +/- HLH protocol. The presence of lymphoma needs surgery and chemoradiation with standard protocol followed by HSCT (71, 72).

In DADA2, management of vasculopathy and stroke is well-established with the use of anti-TNF therapy, however other manifestations including LP, cytopenias, and malignancy need immunosuppressive therapy. Treatment of these includes aggressive chemoradiation with IVIg therapy and TNF blockers to prevent disease progression on case to case basis (73). HSCT is the definitive therapy for the immunological, vascular, and hematological phenotype (74).

No clear treatment protocols for BENTA have been available as of now, however, patients receive multiple therapeutic modalities in the form of steroids, Rituximab, and Sirolimus with variable benefits. Long-term surveillance is important due to the increased risk of B-cell malignancy (75).

STAT3 gain-of-function mutations manifest with a variety of clinical symptoms including lymphoproliferation, and multiorgan autoimmunity. Patients have been treated with Azathioprine and MMF with poor response. Later, IL-6 blockers and HSCT were also tried (76, 77). Recently, the upstream inhibitors, such as Tocilizumab (anti-IL-6 receptor monoclonal antibody) and Janus kinase (JAK) inhibitors are available (78, 79).

## Conclusions

In the patients with IEI, benign/reactive LP must be thoroughly investigated and monitored closely for malignant transformation. Any early-onset nodal or extranodal LP in an appropriate clinical context must be investigated for an underlying IEI. EBV testing must be done routinely in all patients. Flow cytometry and molecular genetics should be used in conjunction with morphology, especially in challenging cases.

## Author Contributions

SSh designed and supervised the manuscript. SSh, RP, GA, MS, KA, RT, MD, and PV reviewed the literature and wrote the manuscript. AR and SSi reviewed and edited the draft. All authors read and approved the final manuscript.

## Conflict of Interest

The authors declare that the research was conducted in the absence of any commercial or financial relationships that could be construed as a potential conflict of interest.

## Publisher’s Note

All claims expressed in this article are solely those of the authors and do not necessarily represent those of their affiliated organizations, or those of the publisher, the editors and the reviewers. Any product that may be evaluated in this article, or claim that may be made by its manufacturer, is not guaranteed or endorsed by the publisher.
