# Supplementary material for: Lymphoproliferation in Inborn Errors of Immunity: The Eye Does Not See What the Mind Does Not Know
Source: Front Immunol. 2022 May 4;13:856601. doi: 10.3389/fimmu.2022.856601 (PMC9114776; doi:10.3389/fimmu.2022.856601)
Supplement: Supplementary file 1 [file Table_1.docx]

**Supplementary Table 1: Flow cytometry panels for diagnosis of LP in IEI**

| **S. No** | **Test/panel** | **Immunophenotypic markers/Methods** |
| --- | --- | --- |
| 1. | B-cell LP | CD19, CD5, CD45, CD20, CD23, CD43, CD79b, CD200, FMC7, CD22, sIg-kappa, sIg-lambda |
| 2. | Plasma cell LP | CD38, CD27, CD19, CD20, CD56, CD117, cyto-kappa and cyto-lambda |
| 3 | T/NK-cell LP | CD3, CD16, CD56, CD45, CD8, CD4, CD7, CD2, CD5, TCRαβ, TCRγδ, CD57, CD10, CD34, TdT, HLA-DR, CD1a, CD52, cyto-TCL1 |
| 4 | Detection of Epstein-Barr virus and telomere length | FLOW-FISH: EBV-encoded small RNA (EBER) probe, CD3+ CD4- CD8- TCRγδ^+^ T-cells |
| 5 | Assessment of clonality | TCR-Vβ repertoire assay; kappa/lambda ratio by FCM |

BL: Burkitt lymphoma; DLBCL: Diffuse large B-cell lymphoma; NGS: Next-generation Sequencing; FCM: Flow Cytometry; FISH: Fluorescence in-situ hybridization; TCR: T-cell receptor
